# 

**Published:** 1916-07-22

**Authors:** 


### A Valuable Committee.

As the result of a recent matinée, the Leicester Royal Infirmary Matinée Committee were able to raise the sum of £229 14s. 6d., and a deputation waited upon the infirmary and handed to Mr. T. Fielding Johnson, jun., the chairman of the board, a cheque for the amount. The work of the Matinée Committee was deservedly eulogised by Mr. T. Fielding Johnson, who acknowledged the institution's great indebtedness to Mr. Oswald Stoll for lending the theatre and staff and providing artistes free of cost to the institution, and to the officers and committee of the matinée for their public-spirited efforts. A sum of £1,416 had been raised during the past ten years, a record upon which the zealous and hard-working Matinée Committee could look back with great satisfaction. Three matinées had, in addition, been arranged for the entertainment of wounded soldiers, by means of which cheer and joy had been brought to over 3,000 soldiers. Mr. Arthur Baum is chairman; Mr. G. Somerville, hon. treasurer; and Mr. J. H. Ward and Mr. Trueman Towers, hon. secretaries of this useful committee.

### HOSPITAL RESULTS IN 1915.

**Gloucestershire Royal Infirmary.**—The bed capacity of the infirmary is 140, but the daily average occupied during 1915 was 86. Nevertheless the number of in-patients is recorded as "the largest that have ever been treated in the wards, being 300 more than the normal in recent years." Ordinary income, £6,407; ordinary expenditure, £8,272. Annual subscriptions are £30 more; 201 military in-patients and 509 military out-patients were treated last year.

**Merthyr General Hospital.**—The hospital is generally admitted to be too small for the district it serves, and an enlargement scheme is under consideration. The accounts are not kept on the Uniform System, and are consequently difficult to understand. We are told that the receipts for 1915 were £4,038, and the expenditure £3,271.

**Monkwearmouth and Southwick Hospital, Sunderland.**—The hospital is largely managed by a committee of representatives of the work-people of this industrial neighbourhood, who supply about one-third of the necessary funds for maintenance. Total income (including £150 legacies), £3,613; total expenditure, £2,885.

**Queen Charlotte's Lying-in Hospital.**—There were 1,817 patients delivered at the hospital and 2,223 in their own homes. The ordinary income was £6,452, and the ordinary expenditure £7,562. For the second year in succession there were no receipts from legacies.

**Royal National Hospital for Consumption, Ventnor.**—The number of patients received was about the same as in the previous year. Some soldiers invalided home, suffering from consumption, have been admitted Of 491 completed cases, 338 improved, the condition of 104 was unchanged or did not improve, and eleven died. The total income (including £2,100 legacies) was £17,967, and the total expenditure £15,458. Annual subscriptions decreased by £157.

**Tunbridge Wells Homœopathic Hospital.**—Subscriptions have fallen in the past year from £251 to £169, and donations from £560 to £345. The total income in 1915 (including £150 legacies) was £1,229 and the expenditure £1,296.

**Royal Victoria Infirmary, Newcastle-on-Tyne.**—£24,722 was raised last year by workpeople and employees, as against £20,756 in 1914. A daily average of 171 beds have been occupied by naval and military patients. The number of new out-patients has fallen from 60,003 to 45,585. The total income (including £1,556 legacies and £9,243 for soldiers) was £51,411, and the total expenditure £46,099. His Majesty the King visited the infirmary on May 20, 1915.

### London County Council.

### Lying-in Homes.

Last quarter the L.C.C. granted twenty-six applications for the registration of lying-in homes. One applicant was notified of the Council's intention to make an order refusing registration, and one person registered has been notified of a proposal to cancel the registration. The grounds of the action were in each case communicated to the person concerned. In several cases applicants were informed of the Council's requirements as regards alterations or repairs to premises, which must be complied with before the application can be granted.

### Massage Establishments.

The L.C.C. has granted 178 applications for the registration, under the new Act, of persons or companies carrying on establishments for massage or special treatment. The number of establishments so registered up to June 30 is therefore 745. Last quarter orders refusing registration were served in two cases, making seventeen in all. Of the three appeals which were pending at the end of the previous quarter, two were dismissed, one with twenty-five guineas costs and the other with ten guineas costs, while the third was abandoned.

### Passing Events and Topics.

### A Hospital Honours a Hero.

A Brass Memorial Tablet to John Travers Cornwell, the sixteen-year-old hero whose name was immortalised in Sir John Jellicoe's Jutland battle dispatch, is to be erected by the Management Committee in the ward in the Grimsby Hospital where he died within twenty-four hours of admission.

### A Tribute to a V.A.D. County Director.

Mr. A. W. Faire, County Director of the Leicester Voluntary Aid Detachment, was presented with cheque for £250 as a contribution to the funds of the Detachment, and an illuminated address at a recent meeting of the Leicester Chamber of Commerce, in recognition of his work in connection with the transport of wounded soldiers reaching Leicester from the Front. In our issue of April 17, 1915, we published an interesting account by Mr. Faire of the organisation of a Voluntary Aid Detachment and the work done by the Leicester ambulance fleet up to that time; this important work has been steadily continuing ever since, and has proved of the greatest value to the authorities.

### Matrons' and Sisters' Appointments.

Hawarden District Council Isolation Hospital, Pentrobin.—Miss R. Stewart has been appointed matron. She was trained at the Leeds Union Infirmary and the Bradford City Hospital. She has since been sister at the Bristol City Hospital, at the Nottingham City Hospital, at the Crewe Borough Hospital; and district and school nurse at Broseley Hospital, Shropshire.

Skipton-in-Craven Joint Infectious Hospital. — Miss Edith Cutter has been appointed matron. She was trained at the Bethnal Green Infirmary and the Huddersfield Sanatorium, where she afterwards became ward sister. She has been ward sister at the City Hospital East, Liverpool; night superintendent at the City Hospital North, Liverpool; sister in charge of Hove Sanatorium; and assistant matron of Ladywell Sanatorium, Salford.

Wirksworth Cottage Hospital.—Miss Annie Hardon has been appointed matron. Trained at University College Hospital, she later became ward sister, and has also been night sister at the Devonshire Hospital, Buxton.

Wolverhampton General Hospital.—Miss Margaret Farrar has been appointed matron of the Convalescent Home. She was trained at York County Hospital, where she later held the posts of sister and night superintendent.

Birkenhead Union Infirmary.—Miss Alice Guest has been appointed assistant matron. She was trained at Bagthorpe Infirmary, and has since held appointments at Jessop Hospital, Sheffield, and Spalding Infirmary.

Whipps Cross Infirmary, Leytonstone.—Miss M. E. Jones has been appointed assistant matron. She was trained at the West Bromwich Infirmary, Staffs, and has since been ward sister at the Camberwell and Whipps Cross Infirmaries, also assistant matron at the City of Westminster Infirmary, Fulham Road. She possesses the certificate of the Central Midwives Board.

Birmingham and Midland Hospital for Women.—Miss L. Howells has been appointed sister. She was trained at Birmingham Workhouse Infirmary, and has since done military nursing at the 1st Southern General Hospital. Miss Howells holds the certificate of the Central Midwives Board.

**NOTE.—Particulars of Appointments for insertion in this column will be welcomed.**

### Personalia.

Lieutenant W. Wilson, R.A.M.C., nose, ear, and throat specialist at St. John Street Hospital, Manchester, has been appointed specialist in a similar capacity to the Cairo district, Egypt.

Dr. Francis Stevens, M.A., M.D., medical officer of health for the borough of Camberwell, has been offered the post of medical officer of the Anglo-Belgian Hospital at Calais for six months, and has been permitted by the Camberwell Borough Council to accept the appointment, subject to the approval of the Local Government Board. Dr. E. C. Bousfield has been selected to act as temporary medical officer of health during his absence.

### Gifts to Hospitals.

Under the will of Mr. D. S. Cowans, of West Mains, Auchterhouse, the Dundee Royal Infirmary and the Dundee Victoria Hospital for Incurables become entitled to £500 each.

Miss Jane Hadley, of Wednesbury, who died on March 7, left £3,000 to the West Bromwich and District Hospital, £200 to the Wolverhampton General Hospital, and £100 to the Wednesbury District Nurses' Association.

RATES OF SUBSCRIPTION: Twelve months, 6/6; Six months, 4/-; Three months, 2/-, post free to any address in the United Kingdom. Abroad, Twelve months, 8/8; Six months, 5/-; Three months, 2/6, post free. THE HOSPITAL "Index" to Volume LIX., October 1915 to March 1916, is now ready, price 1½d. per copy, post free. Address The Manager, "THE HOSPITAL," The Hospital Building, 28/29 Southampton Street, Strand, London, W.C.

